# Heavy menstrual bleeding in women with a bleeding disorder of unknown cause: a common symptom impacting daily life functioning

**DOI:** 10.1016/j.rpth.2026.106950

**Published:** 2026-09-08

**Authors:** Caroline M.A. Mussert, Floor Derikx, Amaury L.L. Monard, Bram Brandt, Sanne H.J. Tegels, Inge Merry, William P.T.M. van Doorn, Laurens Nieuwenhuizen, Marjon H. Cnossen, Yvonne M.C. Henskens, Erik A.M. Beckers, Floor C.J.I. Heubel-Moenen

**Affiliations:** 1 Department of Pediatric Hematology and Oncology, Erasmus MC Sophia Children’s Hospital, University Medical Center Rotterdam, Rotterdam, the Netherlands; 2 Department of Internal Medicine—Hematology, Maastricht University Medical Center +, Maastricht, the Netherlands; 3 Hemophilia Treatment Centre Nijmegen–Eindhoven–Maastricht, Nijmegen, the Netherlands; 4 CARIM—School for Cardiovascular Disease, Maastricht University, Maastricht, the Netherlands; 5 Central Diagnostic Laboratory, Maastricht University Medical Center +, Maastricht, the Netherlands; 6 Department of Hematology, Maxima Medical Center, Veldhoven, the Netherlands

**Keywords:** blood coagulation disorder, female, hemostasis, menorrhagia, quality of life

## Abstract

**Background:**

Persons with an increased bleeding tendency without abnormalities in standard hemostasis tests are classified as having a bleeding disorder of unknown cause (BDUC). The majority are female, and the most common bleeding symptoms are mucocutaneous, including heavy menstrual bleeding (HMB). However, knowledge on HMB in BDUC and the impact on daily functioning is limited.

**Objectives:**

Investigate the prevalence of HMB and HMB-related quality of life (QoL) in women with BDUC, compared with women with mild bleeding disorders (MBDs) and no bleeding disorder (NBD).

**Methods:**

Data from women included in the Maastricht Predictors of Bleeding Evaluation in Adult Hematologic Patients with Bleeding Tendencies study were used. HMB was defined as a score ≥1 on the menorrhagia subdomain of the International Society on Thrombosis and Hemostasis Bleeding Assessment Tool. HMB-related QoL was evaluated using the Menorrhagia Multi-Attribute Scale (MMAS).

**Results:**

In total, 317 women were included (BDUC *n* = 157, MBD *n* = 104, NBD *n* = 56), and 144 women completed the MMAS. HMB was significantly more prevalent in BDUC and MBD (82.8% and 71.2%) compared with NBD (39.3%); *P* < .001. Median total MMAS scores were lower in BDUC and MBD compared with NBD (median [IQR]: 49.8 [34.8-82.6], 54.9 [37.0-75.3], and 74.3 [IQR 46.1-100.0], *P* = .249). Across all groups, women with HMB had significantly lower median total MMAS scores compared with women without HMB (*P* = .002, *P* = .001, and *P* = .002). A higher International Society on Thrombosis and Hemostasis Bleeding Assessment Tool menorrhagia score was associated with a lower MMAS total score.

**Conclusion:**

HMB is highly prevalent among women with BDUC, and HMB substantially impairs HMB-related QoL. This underscores the need for increased clinical awareness of the impact of HMB in this population and for targeted clinical guidelines and optimized management strategies.

## Essentials

• Knowledge on heavy menstrual bleeding (HMB) in bleeding disorder of unknown cause (BDUC) is limited.
• This study evaluates HMB and HMB-related quality of life in women included in the Maastricht bleeding cohort.
• HMB is highly prevalent among women with BDUC and impairs HMB-related quality of life.
• HMB in women with BDUC requires greater clinical attention and targeted management strategies.

## 1 Introduction

In 50% to 75% of persons who are referred to a hemostasis expert for investigation of a clinically relevant bleeding tendency exhibit, no identifiable biological abnormality despite comprehensive laboratory evaluation [[1], [2], [3]]. Subsequently, these persons are categorized as having a bleeding disorder of unknown cause (BDUC). The bleeding phenotype of persons with BDUC is similar to persons with inherited mild bleeding disorders (MBDs) such as von Willebrand disease (VWD), platelet function disorders, and mild coagulation factor deficiencies [2], and is mainly characterized by mucocutaneous bleeding, for example, easy bruising and epistaxis, as well as bleeding around medical and dental procedures [1,2,[4], [5], [6], [7]]. Furthermore, as in women with an inherited MBD [8,9], heavy menstrual bleeding (HMB) is highly prevalent among women with BDUC, with frequencies ranging between 60% and 90%, as well as postpartum hemorrhage in 30% to 65% [1,2,4,5]. This high proportion of women may be explained by the sex-specific challenges experienced by women, such as menstruation and childbirth, prompting referral and investigation of bleeding symptoms. Strikingly, over 60% of persons with BDUC are women, and diagnosis is indeed most common during the reproductive years, between 30 and 40 years of age [1,2,4,7].

HMB is historically defined as menstrual blood loss exceeding 80 mL [10]. However, as methods to accurately measure menstrual blood loss to determine HMB are often inconvenient and time-consuming, the National Institute for Health and Care Excellence redefined HMB as “excessive menstrual blood loss which interferes with a woman’s physical, social, emotional and/or material quality of life (QoL)” [11]. Recent studies have increasingly focused on the impact of menstruation and childbirth in women with inherited bleeding disorders and demonstrated that HMB negatively influences QoL [[12], [13], [14], [15]]. In a recent survey conducted by the European Haemophilia Consortium, HMB was reported as the bleeding symptom with the greatest impact on daily life [12]. HMB can affect physical health, psychosocial well-being, and sexual functioning, as well as reproductive decisions. Furthermore, HMB is associated with iron deficiency and anemia [16,17], which may subsequently result in fatigue, dizziness, and poor concentration [13,17], thereby negatively influencing daily life activities and educational or occupational performance.

Nevertheless, the impact of HMB on QoL in women with BDUC remains unknown, and the absence of a clear pathophysiological mechanism behind the bleeding tendency further complicates gynecological management in this population. While clear guidelines for the gynecological management of females with inherited bleeding disorders, such as VWD, are available [18,19], optimal strategies for managing HMB and childbirth in women with BDUC have not yet been established [17,20]. Therefore, the aim of this study is to investigate the prevalence of HMB and HMB-related QoL in women with BDUC, compared with women with MBDs and no bleeding disorder (NBD) included in the Predictors of Bleeding Evaluation in Adult Hematologic Patients with Bleeding Tendencies (ProBe-AHP) study.

## 2 Materials and Methods

### 2.1 Study design and study population

Clinical and laboratory data from the ongoing ProBe-AHP study, conducted at the Maastricht University Medical Centre+, the Netherlands, were used for this analysis. The ProBe-AHP study is an observational study among adult patients referred to a hemostasis expert for investigation of a (suspected) bleeding disorder. Exclusion criteria included pregnancy or lactation, active bleeding due to surgical or obstetrical causes, and the inability to discontinue medication, herbal preparations, or supplements influencing hemostasis. Following laboratory evaluation and discussion of results within the multidisciplinary team specializing in hemostasis, patients were stratified into 3 groups: diagnosed MBD, NBD, or BDUC. BDUC was defined by the absence of abnormalities in standard hemostasis tests (complete blood count, MPV, PT, activated partial thromboplastin time, thrombin time, fibrinogen; von Willebrand factor activity and antigen; coagulation factors [F]II, FV, FVII, FVIII, FIX, FX, FXI, and FXIII; Platelet Function Analyzer-200 and Light Transmission Aggregometry) combined with either an abnormal International Society on Thrombosis and Hemostasis Bleeding Assessment Tool (ISTH-BAT) score (≥4 in men, and age-adjusted reference ranges for women: 18-30 years: 0-4; 31-51 years: 0-5; 52-88 years: 0-6) [21] or observations of abnormal or atypical bleeding phenotype based on *clinical gestalt*. Diagnostic criteria for MBD were based on national and international guidelines [[22], [23], [24], [25], [26]], and are presented in Supplementary Table 1 [27]. NBD was defined as normal hemostasis tests and a normal bleeding phenotype based on ISTH-BAT and *clinical gestalt*. The study design and patient inclusion have previously been described in more detail in prior reports [22]. For this analysis, only women were included who were enrolled in the study between April 1, 2014 and December 31, 2025, and had reached a final diagnosis. An overview of the patient inclusion process is presented in Figure 1. The ProBe-AHP study was approved by the Medical Ethics Research Committee of the Maastricht University Medical Center, Maastricht, the Netherlands (MEC-14-4-036, NL9643) and is registered at the Dutch Trial Register (NL9643). All patients signed informed consent to use data for research purposes.

**Table 1:** Baseline characteristics.

	BDUC	MBD	NBD	*P*
**Number**	157 (49.5%)	104 (32.8%)	56 (17.7%)	
**Age at enrollment, y**	43 ± 15.7	42 ± 17.5	49 ± 19.1	*P* = .043
**Referred by gynecologist**	16/157 (10.2%)	10/104 (9.6%)	3/56 (5.4%)	*P* = .549
**HMB reason for referral**	11/157 (7.0%)	7/104 (6.7%)	3/56 (5.4%)	*P* = .912
**ISTH-BAT score**	8.55 ± 3.39	7.80 ± 4.57	3.13 ± 1.84	*P* < .001 *BDUC vs MBD: P = .327* *BDUC vs NBD: P < .001* *MBD vs NBD: P < .001*
**% abnormal ISTH-BAT score**	130/157 (82.8%)	69/104 (66.3%)	1/56 (1.8%)^a^	*P* < .001 *BDUC vs MBD: P = .002* *BDUC vs NBD: P < .001* *MBD vs NBD: P < .001*
**Hemoglobin (mmol/L)**	8.38 ± 0.66	8.36 ± 0.69	8.46 ± 0.60	*P* = .669
**Anemia**	11/156 (7.1%)	10/101 (9.9%)	2/56 (3.6%)	*P* = .340
**Blood group O**	70/152 (46.1%)	55/95 (57.9%)	28/55 (50.9%)	*P* = .194

Data are presented as mean ± SD or number (%)

BDUC, bleeding disorder of unknown cause; DOAC, direct oral anticoagulant; HMB, heavy menstrual bleeding; ISTH-BAT, International Society on Thrombosis and Hemostasis Bleeding Assessment Tool; MBD, mild bleeding disorder; NBD, no bleeding disorder.

a Patient with an elevated ISTH-BAT score who had received treatment with a DOAC in the past. Given that the bleeding symptoms had decreased after discontinuation of the DOAC and laboratory results were within normal ranges, this patient was classified as having NBD.

**Figure 1:**
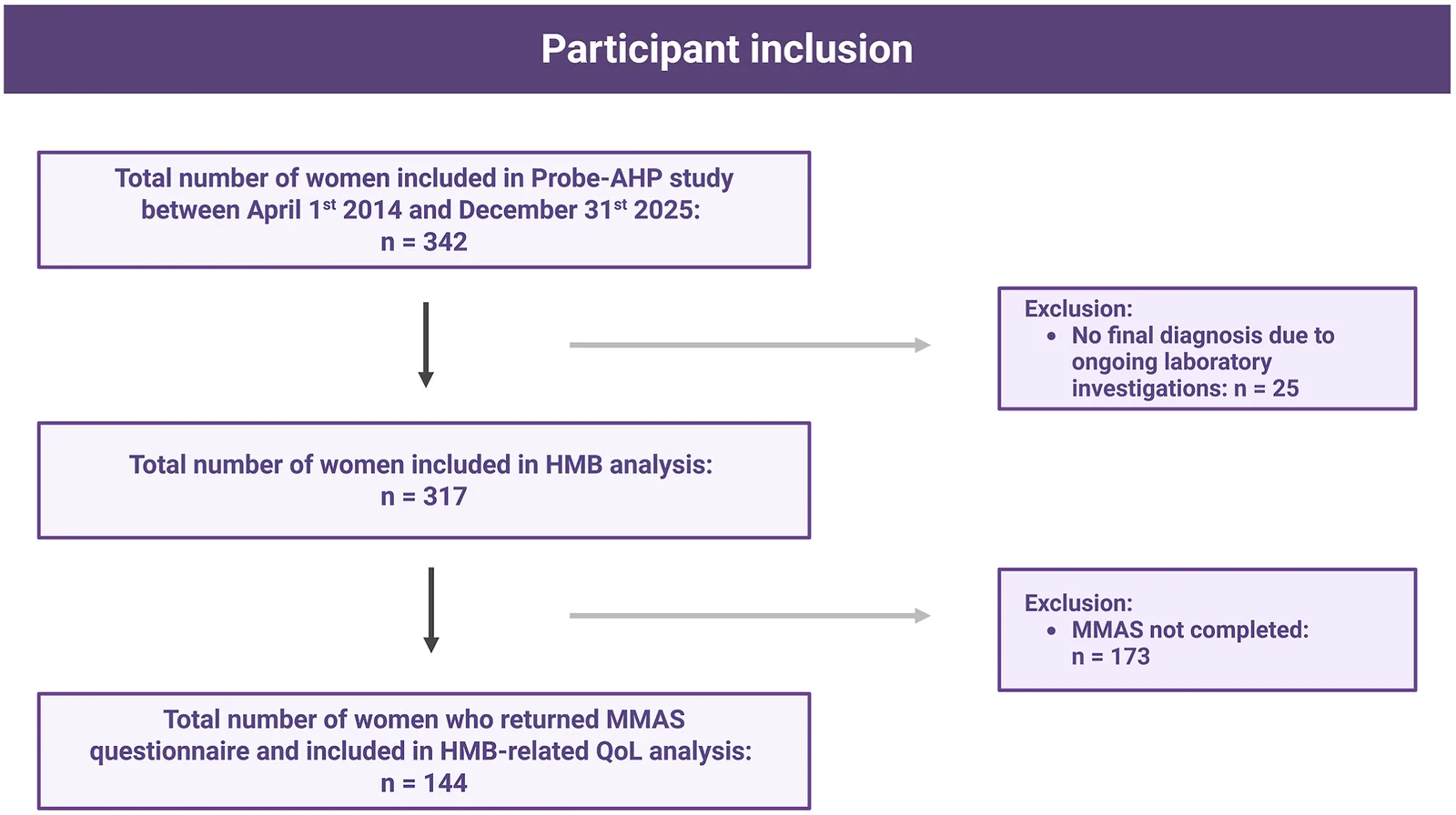
Flow diagram of participant inclusion. Predictors of bleeding evaluation in adult hematologic patients with bleeding tendencies; HMB, heavy menstrual bleeding; MMAS, Menorrhagia Multi-Attribute Scale; Probe-AHP, predictors of bleeding evaluation in adult hematologic patients with bleeding tendencies. *Source:* Created in BioRender. Cnossen M. (2026) https://BioRender.com/fq9icka.

### 2.2 Assessments

Patients with a (suspected) bleeding disorder were investigated on an outpatient clinic basis according to the standard hemostasis workup of the Maastricht University Medical Center. During this visit, blood was withdrawn to perform both standard and experimental laboratory hemostasis tests, and clinical data were collected. The ISTH-BAT was used to assess the bleeding phenotype. The ISTH-BAT consists of 14 domains measuring different bleeding symptoms, with a score ranging from 0 (no or trivial symptoms) to 4 (severe symptoms requiring substantial treatment or intervention). The total ISTH-BAT score is calculated by adding up the individual domain scores. Age-adjusted reference ranges for women were used as cut-off values [21]. The ISTH-BAT was administered by a nurse practitioner or by 1 of 4 hematologists. The menorrhagia component of the ISTH-BAT was used to determine the presence of HMB. HMB was defined as a score of ≥1 on the ISTH-BAT subdomain [9]. The total score in this subdomain is based on the age of onset, severity, and treatment of HMB and ranges from 0 (no or trivial symptoms) to 4 (severe symptoms requiring, eg, blood transfusion, replacement therapy, or endometrial ablation) [28].

In addition, all included women were asked to complete the Menorrhagia Multi-Attribute Scale (MMAS), a validated tool to assess HMB-related QoL [29]. The MMAS is a questionnaire that subjectively measures the influence of HMB on 6 different domains: practical difficulties, social life, psychological well-being, physical health, work/daily routine, and family life. Each domain consists of 4 statements that respondents can choose from to indicate the influence HMB has on that specific domain (Supplementary Table 2). The total MMAS score is calculated by adding up the weighted scores, as defined in the original study [30], of the selected statements from each domain, resulting in a multiattribute utility score ranging from 0 to 100, with lower scores indicating poorer HMB-related QoL. In menopausal women, the ISTH-BAT menorrhagia subdomain and MMAS scores were based on menstruations in the past.

### 2.3 Statistical analysis

Statistical analyses were performed with IBM SPSS Statistics version 28 and visualized using GraphPad Prism 5. Descriptive statistics were used to summarize the data (numbers and percentages). Continuous variables were expressed as mean ± SD for normally distributed data or as median and interquartile range for nonnormally distributed data, based on visual representation and normality tests. Categorical variables were presented as counts (n) and percentages. Group comparisons were performed to examine differences between persons with BDUC, MBD, and NBD. One-way analysis of variance (anova) was used for multiple group comparisons with normally distributed continuous data, while for nonnormally distributed continuous data, the Kruskal–Wallis test was used. For categorical data, differences between groups were tested using the chi-squares test.

For normally distributed data, post hoc comparisons were performed using Games–Howell for continuous data or a pairwise chi-squared test for categorical data. For nonnormally distributed data, the Mann–Whitney U test was performed. Bonferroni correction was applied to account for multiple testing. Cliff’s Delta was used to measure effect size. A 2-way anova was used to assess the effect of HMB and disease group on the total MMAS score. Linear regression analysis was used to evaluate the association between HMB severity (ISTH-BAT score of menorrhagia subdomain) and MMAS score. Statistical significance was defined as a *P* value < .05 (*P* < .05).

## 3 Results

### 3.1 Baseline characteristics

In total, 317 of 342 women enrolled in the ProBe-AHP study were included in the analysis. A total of 25 women were excluded as laboratory investigations were still ongoing (Figure 1). Following clinical and laboratory workup, 157 (49.5%) women were diagnosed with BDUC, 104 (32.8%) with MBD, and 56 (17.7%) with NBD (Table 1). Ages at study inclusion ranged between 18 and 91 years, with mean ages of 43 years (SD 15.7) in BDUC, 42 years (SD 17.5) in MBD, and 49 years (SD 19.1) in NBD (*P* = .043). 10.2% of women with BDUC were referred by a gynecologist for evaluation of bleeding symptoms (mean age 33 years, age range 18-52 years), 9.6% of women with MBD, and 5.4% with NBD. Specifically, HMB was the reason for referral in 11 (7.0%), 7 (6.7%), and 3 (5.4%) women.

Women with BDUC and MBD demonstrated similarly high total ISTH-BAT scores (8.55 ± 3.39 and 7.80 ± 4.57), both significantly higher than those observed in women with NBD (3.13 ± 1.84); *P* < .001 (Table 1). According to the new age-adjusted reference ranges for ISTH-BAT cut-off values, a significantly larger proportion of women with BDUC and MBD had abnormal total ISTH-BAT scores (82.8% and 66.3%, respectively) compared with women with NBD (1.8%); *P* < .001. Consequently, 17.2% of women with BDUC had a normal ISTH-BAT score and were diagnosed with BDUC based on *clinical gestalt*.

No significant differences were observed in hemoglobin levels and the prevalence of anemia among women with BDUC, MBD, and NBD (Table 1). Mean hemoglobin levels in BDUC were within the normal range, and the prevalence of anemia was low across all groups. Subgroup analysis stratified by presence of HMB similarly revealed no significant differences in hemoglobin levels (BDUC: *P* = .812; MBD: *P* = .920; NBD: *P* = .374) or anemia prevalence (BDUC: *P* = .455; MBD: *P* = .117; NBD: *P* = .752). The distribution of blood group O was comparable across groups, and no significant differences were observed between women with and without HMB (BDUC: *P* = .853; MBD: *P* = .225; NBD: *P* = .200).

### 3.2 HMB prevalence and treatment

First, the prevalence and severity of HMB across disease groups were assessed. HMB was highly prevalent among women with BDUC and MBD (82.8% and 71.2%, respectively) and significantly more common compared with NBD (39.3%); *P* < .001 (Figure 2A). When applying an ISTH-BAT menorrhagia subdomain score of ≥2 to define HMB, HMB was still significantly more prevalent in women with BDUC and MBD (63.1% and 56.7%), compared with NBD (30.4%); BDUC − MBD: *P* = .306; BDUC − NBD: *P* < .001; MBD − NBD: *P* = .001.

**Figure 2:**
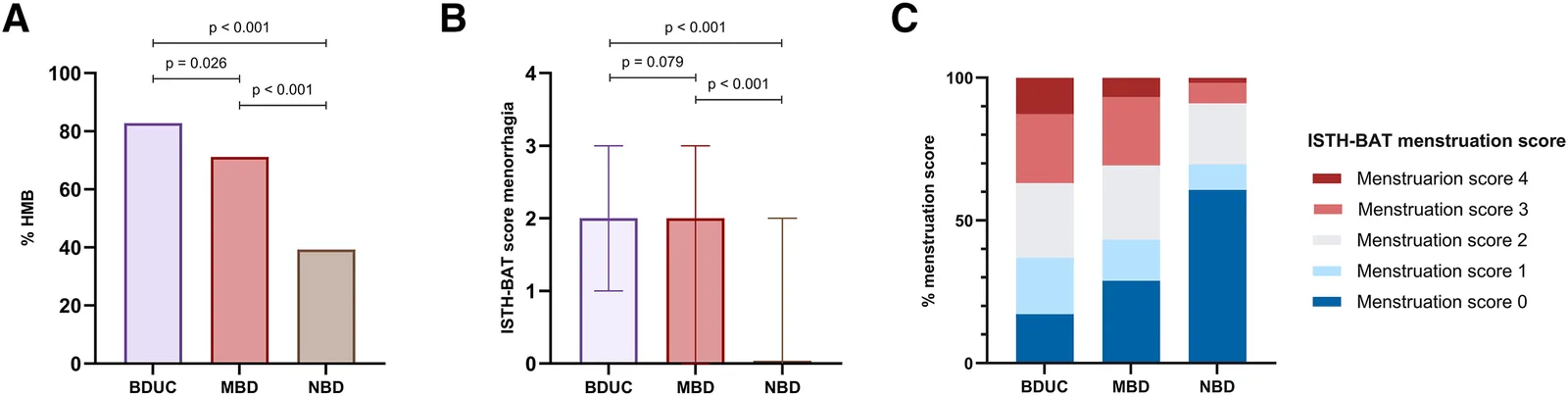
Prevalence and severity of heavy menstrual bleeding (HMB). (A) Percentage of women with HMB in bleeding disorder of unknown cause (BDUC), mild bleeding disorder (MBD), and no bleeding disorder (NBD). HMB was defined as a score of ≥1 on the menorrhagia subdomain of the International Society on Thrombosis and Hemostasis Bleeding Assessment Tool (ISTH-BAT). This domain score ranges from 0 to 4 with higher scores indicating more severe symptoms requiring substantial treatment or intervention. (B) ISTH-BAT menorrhagia subdomain scores, demonstrating HMB severity. Data is presented as median with interquartile range. (C) Overview of ISTH-BAT menorrhagia subdomain scores. Score 0: no/trivial symptoms; score 1: consultation only, changing pads more frequently than every 2 hours, clot and flooding, or pictorial blood loss assessment chart score >100; score 2: time off work/school >2/year, requiring antifibrinolytics, or hormonal or iron therapy; score 3: requiring combined treatment with antifibrinolytics and hormonal therapy or present since menarche and >12 months; score 4: acute menorrhagia requiring hospital admission and emergency treatment, requiring blood transfusion, replacement therapy, desmopressin, or requiring dilatation and curettage or endometrial ablation or hysterectomy.

Based on the ISTH-BAT menorrhagia subdomain, women with BDUC and MBD experienced more severe menstrual bleeding than those with NBD, with median menorrhagia subdomain scores of 2.0 (IQR 1.0-3.0) for BDUC, 2.0 (IQR 0.0-3.0) for MBD, and 0.0 (IQR 0.0-2.0) for NBD; *P* < .001 (Figure 2B, C). Among women reporting HMB, however, median subdomain scores did not significantly differ across groups (BDUC: 2.0 [IQR 2.0-3.0]; MBD: 2.0 [IQR 2.0-3.0]; NBD: 2.0 [IQR 2.0-2.0]; *P* = .359).

Significantly more women with HMB used oral contraception compared with women without HMB across all groups (BDUC: HMB 91/119 [76.5%] vs no HMB 9/18 [50%]; *P* = .018; MBD: HMB 48/64 [75%] vs no HMB 6/19 [31.6%]; *P* < .001; NBD: HMB 15/21 [71.4%] vs no HMB 10/26 [38.5%]; *P* = .024) (Figure 3A). Regardless of the diagnosis, a large proportion of women with HMB used oral contraception (*P* = .879) predominantly as treatment for HMB (BDUC: 84/91 [92.3%]; MBD: 45/48 [93.4%]; NBD: 14/15 [93.3%]; *P* = .973). However, the use of tranexamic acid (TXA) as treatment for HMB was markedly low across all groups (Figure 3B). Only a small proportion of women with HMB received TXA (BDUC: 6/91 [6.6%]; MBD: 3/48 [6.3%]; NBD: 0/15 [0%]). Similarly, the use of iron supplementation was limited (BDUC: 5/91 [5.5%]; MBD: 1/48 [2.1%]; NBD: 1/15 [6.7%]).

**Figure 3:**
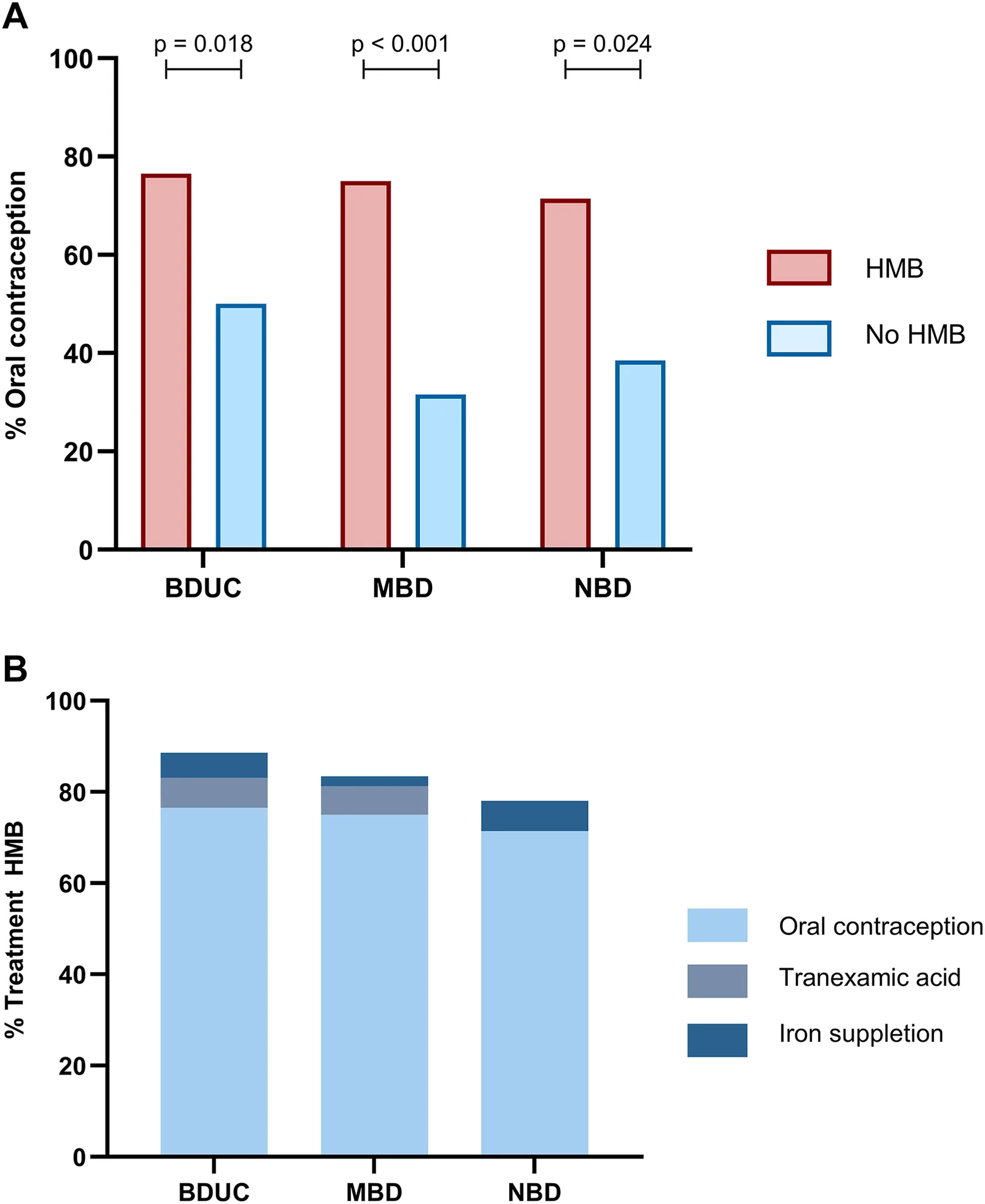
Used treatment for heavy menstrual bleeding (HMB). (A) Oral contraception use in women with HMB compared with women without HMB in bleeding disorder of unknown cause (BDUC), mild bleeding disorder (MBD), and no bleeding disorder (NBD). (B) Percentage of women with HMB who received oral contraception, tranexamic acid or iron supplementation as treatment for HMB.

### 3.3 HMB-related QoL

#### 3.3.1 MMAS score

The MMAS score was used to assess the impact of HMB on QoL. Of the 317 women included in the analysis, 144 MMAS questionnaires were returned (45.4%), of which 72 women had BDUC (50%), 50 women had MBD (34,7%), and 22 had NBD (15.3%) (Table 2). Median total MMAS scores were lower in women with BDUC and MBD (49.8 [IQR 34.8-82.6] and 54.9 [IQR 37.0-75.3], respectively) than in those with NBD (74.3 [IQR 46.1-100.0]) (Figure 4A). However, no statistically significant difference was found (*P* = .249).

**Table 2:** Total MMAS and subdomain scores in BDUC, MBD, and NBD.

	BDUC			MBD			NBD		*P*
**Number of completed MMAS questionnaires**	72/157 (45.9%)			50/104 (48.1%)			22/56 (39.3%)		
**MMAS total score**	49.8 [34.8-82.6]			54.9 [37.0-75.3]			74.3 [46.1-100.0]		.249
MMAS subdomain scores Q1: practical difficulties Q2: social life Q3: psychological well-being Q4: physical health Q5: work/daily routine Q6: family life	3.1 [0.0-9.4] 6.7 [2.7-10.0] 8.4 [2.9-14.0] 4.2 [4.2-14.9] 11.3 [4.1-18.0] 14.0 [14.0-23.0]			6.3 [3.1-9.4] 6.7 [2.7-10.0] 8.4 [2.9-14.0] 4.2 [4.2-14.9] 11.3 [4.1-18.0] 14.0 [14.0-23.0]			9.4 [3.1-14.0] 6.7 [6.7-10.0] 11.2 [2.9-14.0] 14.9 [4.2-21.0] 18.0 [11.3-18.0] 23.0 [5.3-23.0]		.027 .281 .800 .734 .096 .763
	**HMB *n* = 59**	**No HMB *n* = 13**	***P***	**HMB *n* = 41**	**No HMB *n* = 9**	***P***	**HMB *n* = 11**	**No HMB *n* = 11**	***P***
**MMAS total score**	43.7 [31.6-74.9]	79.7 [64.7-100.0]	.002	46.8 [36.5-64.3]	95.4 [82.6-100.0]	.001	46.1 [19.2-70.6]	89.3 [80.1-100.0]	.002
MMAS subdomain scores Q1: practical difficulties Q2: social life Q3: psychological well-being Q4: physical health Q5: work/daily routine Q6: family life	3.1 [0.0-9.4] 6.7 [2.7-10.0] 8.4 [2.9-14.0] 4.2 [4.2-14.9] 11.3 [4.1-14.7] 14.0 [5.3-23.0]	9.4 [9.4-14.0] 10.0 [6.7-10.0] 14.0 [8.4-14.0] 21.0 [11.3-21.0] 11.3 [11.3-18.0] 23.0 [14.0-23.0]	.002 .054 .014 .003 .029 .081	3.1 [0.0-9.4] 6.7 [2.7-6.7] 8.4 [2.9-14.0] 4.2 [4.2-14.9] 4.1 [4.1-11.3] 14.0 [14.0-23.0]	9.4 [9.4-14.0] 10.0 [10.0-10.0] 14.0 [14.0-14.0] 21.0 [14.9-21.0] 18.0 [18.0-18.0] 23.0 [23.0-23.0]	.015 .003 .008 .005 .008 .051	3.1 [0.0-9.4] 6.7 [4.8-8.4] 2.9 [2.9-11.2] 4.2 [0.0-14.9] 11.3 [2.1-18.0] 14.0 [2.7-23.0]	14.0 [11.7-14.0] 10.0 [6.7-10.0] 14.0 [11.2-14.0] 21.0 [9.6-21.0] 18.0 [18.0-18.0] 23.0 [23.0-23.0]	.001 .065 .023 .016 .034 .047

Data are presented as median [IQR].

BDUC, bleeding disorder of unknown cause; HMB, heavy menstrual bleeding; MBD, mild bleeding disorder; MMAS, Menorrhagia Multi-Attribute Scale; NBD, no bleeding disorder.

**Figure 4:**
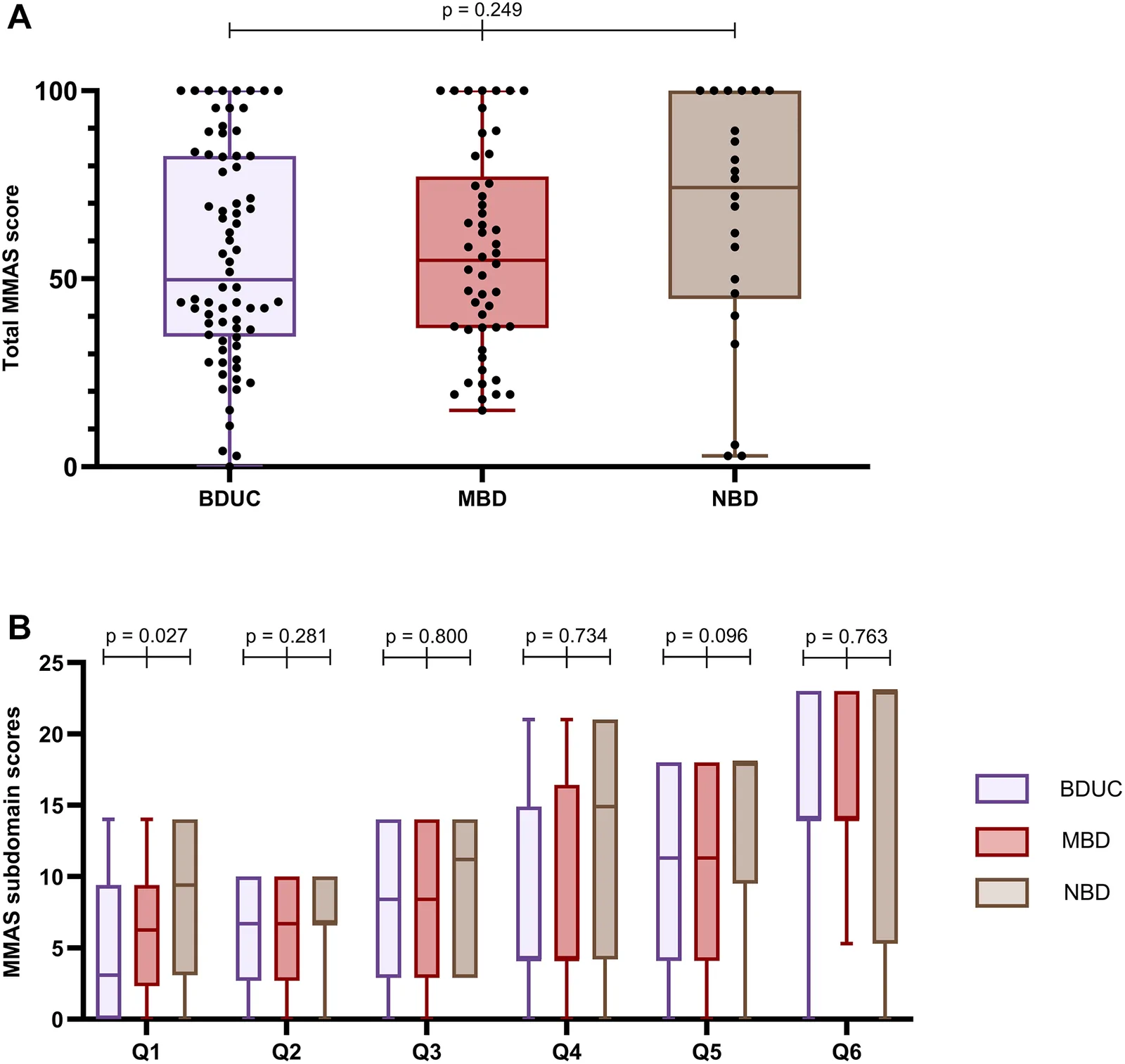
Menorrhagia Multi-Attribute Scale (MMAS) scores. (A) Total MMAS scores in bleeding disorder of unknown cause (BDUC), mild bleeding disorder (MBD), and no bleeding disorder (NBD). Data are presented as individual total MMAS scores, with boxplots representing medians with interquartile range, and bars representing minimum and maximum score. The total MMAS score is calculated by summing the weighted scores of the subdomains, resulting in a multiattribute utility score ranging from 0 to 100, with lower scores indicating poorer HMB-related quality of life. (B) MMAS subdomain scores in BDUC, MBD, and NBD. Data are presented as median with interquartile range. Q1, practical difficulties; Q2, social life; Q3, psychological well-being; Q4, physical health; Q5, work/daily routine; and Q6, family life. Q, question.

Within the NBD group, 3 outliers were observed with total MMAS scores of 2.9, 2.9, and 5.6. These 3 women had ISTH-BAT menorrhagia subdomain scores of 3, 2, and 2, respectively, and each scored one point across 2 other domains (epistaxis, cutaneous bleeding, and/or oral cavity bleeding). The woman with a menorrhagia subdomain score of 3 experienced menstrual periods lasting 6 to 7 days, during which she was confined to her home for the first 3 days, requiring hourly sanitary pad changes, and reported frequent overflow.

Evaluation of the individual MMAS subdomains demonstrated a consistent pattern in which women with BDUC and MBD scored lower across all subdomains except the influence of HMB on social life (Table 2, Figure 4B). A statistically significant difference was only observed for the practical difficulties subdomain, and post hoc analysis showed that this difference was limited to the comparison between BDUC and NBD (*P* = .008). Overall, these findings suggest that women with BDUC and MBD tend to report a lower HMB-related QoL across most subdomains, with the exception of social functioning, compared with women with NBD.

To validate the observed difference in total MMAS score, we performed a sensitivity analysis by calculating effect sizes using Cliff’s Delta. The comparison between BDUC and MBD demonstrated a negligible effect (*δ* = −0.01), indicating near-complete overlap. Comparisons between BDUC and MBD with NBD showed small effects: BDUC vs NBD (*δ* = −0.23) and MBD vs NBD (*δ* = −0.22). These results indicate that although women with BDUC and MBD reported lower HMB-related QoL than those with NBD, the magnitude of these differences was small.

To investigate potential responder bias related to the severity of HMB, we also compared ISTH-BAT menorrhagia subdomain scores between responders and nonresponders within each disease group. No significant differences were observed in each group (BDUC: *P* = .585; MBD: *P* = .069; NBD: *P* = .213).

#### 3.3.2 Sensitivity analysis of MMAS score

To assess whether the weighted scores from the original MMAS validation study influenced the lack of observed differences between the groups, we performed a sensitivity analysis. In this analysis, the weighted scores were replaced with proportional scores, ensuring that all domains contributed equally to the total score. The statements within each subdomain were assigned an equal score ranging from 0 (statement 1) to 3 (statement 4), resulting in a maximum total score of 18, where higher scores indicate poorer HMB-related QoL. This differs from the original MMAS score system, in which higher scores indicate better QoL.

Nevertheless, results remained consistent with the primary analysis. Women with BDUC and MBD continued to score worse on both the total MMAS score and subdomain scores, with the exception of social life, compared with the NBD group, although these differences were not significantly different (Supplementary Table 3).

#### 3.3.3 MMAS score in women with and without HMB

In addition, MMAS scores were compared between women with and without HMB. Women with HMB had significantly lower median total MMAS scores than those without HMB across all 3 disease groups (BDUC: *P* = .002; MBD: *P* = .001; NBD: *P* = .002) (Figure 5A). Among women with HMB, median MMAS total scores ranged between 43.7 and 46.8, whereas scores among women without HMB ranged from 79.7 to 95.4, indicating a substantial reduction in HMB-related QoL in the presence of HMB.

**Figure 5:**
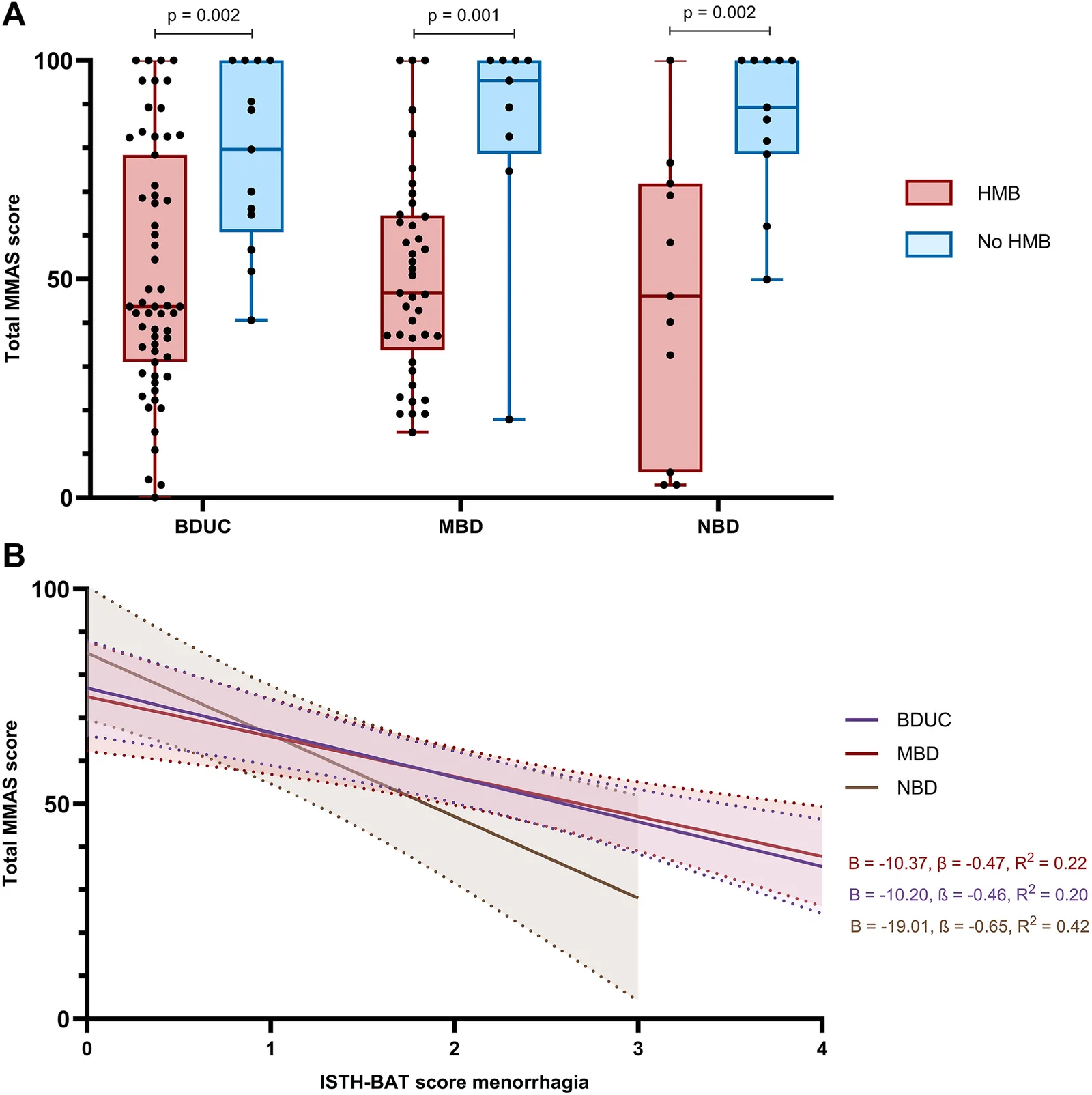
Influence of heavy menstrual bleeding (HMB) on quality of life. (A) Total Menorrhagia Multi-Attribute Scale (MMAS) scores in women with HMB compared with women without HMB in bleeding disorder of unknown cause (BDUC), mild bleeding disorder (MBD) and no bleeding disorder (NBD). Data are presented as individual total MMAS scores, with boxplots representing medians with interquartile range and bars representing minimum and maximum score. (B) Linear regression of the association between the severity of menstrual bleeding, according to the International Society on Thrombosis and Hemostasis Bleeding Assessment Tool (ISTH-BAT) menorrhagia subdomain score, and quality of life, indicated by the total MMAS score. Higher ISTH-BAT menorrhagia score was associated with lower total MMAS score. *R*^2^ indicates goodness-of-fit.

We also compared MMAS scores between women with and without HMB using a stricter definition of HMB, based on an ISTH-BAT menorrhagia subdomain score of ≥2. Consistent with the primary analysis, women with HMB had significantly lower median total MMAS scores than those without HMB across all disease groups (Supplementary Table 4).

Subdomain analysis showed that women with BDUC and MBD consistently reported lower scores across all subdomains, except for the work/daily routine domain in BDUC (Table 2). After Bonferroni correction for multiple testing, the practical difficulties and physical health subdomains remained significantly lower in women with HMB compared with those without HMB in all disease groups.

A 2-way anova was performed to evaluate the effects of HMB and disease group on the total MMAS scores (Supplementary Table 5). HMB had a significant effect on the total MMAS score (*P* < .001), whereas the disease group did not (*P* = .884). No interaction effect was observed between HMB and the disease group (*P* = .574), indicating that the negative impact of HMB on QoL was similar across all disease groups.

### 3.4 Severity of menstrual bleeding and QoL

Linear regression analysis was used to evaluate the association between the severity of menstrual bleeding, according to the ISTH-BAT menorrhagia subdomain score, and QoL. A higher ISTH-BAT menorrhagia score was associated with a lower total MMAS score across all groups: BDUC (*β* = −0.47, *P* < .001 [95% CI, −0.69 to −0.26]), MBD (*β* = −0.46, *P* < .001 [95% CI, −0.74 to −0.21]), and NBD (*β* = −0.65, *P* = .001 [95% CI, −1.36 to −0.39) (Figure 5B). The strongest association was observed in the NBD group (*β* = −0.65), compared with BDUC (*β* = −0.47) and MBD (*β* = −0.46).

## 4 Discussion

This study focused on HMB and its impact on HMB-related QoL among women with BDUC, MBD, and NBD. HMB was highly prevalent in both BDUC and MBD, affecting 83% and 71% of women, respectively. In contrast, HMB was present in 39% of women in the NBD group, a proportion consistent with previously reported prevalence rates among European women without bleeding disorders [31,32]. The frequency of HMB observed in our BDUC cohort aligns with earlier studies reporting frequencies ranging between 60% and 90% [1,2,4,5]. These findings are also comparable with those reported in women with other inherited bleeding disorders, including congenital platelet disorders (87%) [14], VWD (81-93%) [33,34], and rare bleeding disorders (80%) [9].

We observed lower HMB-related QoL among women with BDUC and MBD compared with women with NBD, with median total MMAS scores aligning with scores found in women with congenital platelet disorders [14]. These scores were also lower than those of a group of healthy Japanese women, who had normal menstrual blood loss and a mean MMAS score of 95 ± 8.4 [35]. However, no statistically significant differences were found between disease groups. Within each disease group, women with HMB consistently reported significantly lower HMB-related QoL than women without HMB. However, neither the disease group itself nor the interaction between the disease group and HMB influenced total MMAS scores. This indicates that the negative effect of HMB on QoL we observed is comparable across disease groups and was not determined by the underlying bleeding disorder. These findings suggest that it is primarily the presence of HMB that adversely affects QoL, rather than a BDUC or MBD diagnosis. The absence of significance may be partly attributable to substantial variability in MMAS scores within groups and 3 notable outliers in the NBD group, which may have influenced the results given the small sample size. These outliers, particularly the described women with an ISTH-BAT menorrhagia score of 3, may also introduce some uncertainty regarding potential diagnostic misclassification, especially since BDUC is a diagnosis per exclusion and is based on the BAT score, which is known to have limitations [36]. Therefore, the MMAS may contribute to the classification of patients based on clinical gestalt. Nonetheless, nonhematologic factors can also cause or contribute to HMB and may account for these observations [37]. Importantly, the prevalence of HMB may be influenced by the applied ISTH-BAT threshold. Although we used a menorrhagia subdomain score of ≥1 to define HMB, applying a stricter threshold of ≥2 demonstrated consistent results. The prevalence of HMB remained significantly higher in women with BDUC and MBD compared with NBD, and women with HMB continued to report significantly lower MMAS scores compared with women without HMB across all disease groups.

Besides the association between the presence of HMB and QoL, we also found an association between the severity of HMB and HMB-related QoL across disease groups. Although the regression coefficient appeared larger in women with NBD, this finding should be interpreted with caution due to the few MMAS outliers in the NBD group (data not shown) and limited sample size. One possible explanation for this observation may be that women with (inherited) bleeding disorders are more familiar with bleeding-related symptoms, having experienced them from an early age and in women within the family [38], which may reduce their perceived impact on QoL. In contrast, for women with NBD, HMB may represent an isolated symptom, thereby posing a relatively greater impact.

Regardless of the high prevalence of HMB among women with BDUC and MBD and the well-established association between HMB, anemia, and iron deficiency [16], observed hemoglobin levels in our cohort were within the normal range and did not differ from those observed in women with NBD. The prevalence of anemia was also low across all groups. Iron parameters, however, were not assessed in the ProBe-AHP study. Our findings are consistent with those of Dreier et al. [39], who similarly reported normal hemoglobin levels and no significant differences between women with BDUC, MBD, and healthy controls, along with comparably low rates of anemia. Nevertheless, despite low anemia rates, they observed iron deficiency in 44% of women with HMB and a diagnosis of BDUC or MBD, indicating that iron deficiency without anemia is also common. This observation is supported by other studies demonstrating that anemia is present in only approximately half of women with HMB and iron deficiency [40,41]. Importantly, because iron deficiency can cause symptoms such as fatigue, dizziness, and impaired concentration, it can adversely affect QoL independent of menstrual bleeding episodes and therefore on a daily basis [16]. As a result, even in the absence of anemia, iron deficiency can still negatively influence attendance and performance at school or work. The use of iron supplementation was low. However, as this was documented based on review of the electronic patient record rather than assessed at the time of inclusion, and given that iron is also frequently prescribed by general practitioners, its use is likely to be underrepresented in our study.

Notably, the use of TXA for the management of HMB was markedly low across women with BDUC, MBD, and NBD. This is especially relevant since TXA is a proven effective treatment for reducing menstrual blood loss in women with established inherited bleeding disorders [40,42,43], as well as in idiopathic HMB [44]. Moreover, previous studies have demonstrated that TXA use can improve QoL [42,44]. For women with inherited bleeding disorders specifically, the use of TXA is recommended as supplementary hemostatic treatment, alongside hormonal treatment, during menstruation [17]. Despite these recommendations, optimal gynecological management of HMB in women with BDUC remains insufficiently defined [45], and clear guidelines are currently lacking. Nevertheless, the low utilization of TXA observed in our study highlights an important gap in clinical practice. Given its proven efficacy and beneficial impact on QoL, greater emphasis should be placed on incorporating TXA into the management of HMB among women with BDUC.

Several limitations should be considered. The time interval between inclusion and questionnaire completion varied, as women who had not yet completed the MMAS upon diagnostic assessments were contacted later, sometimes several years after initial enrollment. As a result, a proportion of women had started menopause by the time they completed the questionnaire, leading to retrospective reporting of HMB-related QoL. Additionally, some women were already postmenopausal at the time of inclusion, meaning that their responses also reflected past menstrual experiences. Furthermore, a subset of women had already initiated treatment for HMB at the time of MMAS completion and may consequently have reported less impairment in QoL than they would have prior to treatment, potentially leading to an overestimation of their MMAS scores. Lastly, the response rate of the MMAS questionnaires was 45%. Women experiencing more severe HMB may have been more likely to complete the questionnaire, potentially resulting in an overrepresentation of women with greater bleeding burden among MMAS responders. However, comparison of ISTH-BAT menorrhagia scores between MMAS responders and nonresponders within each disease group did not reveal significant differences. Nevertheless, to our knowledge, this is the first study using the MMAS in women with both BDUC and MBD, thereby providing detailed insights into HMB-specific QoL in women with diagnosed and undiagnosed bleeding disorders. The long inclusion period enabled the inclusion of a relatively large cohort of women, including a comparison group with NBD, all of whom were referred for evaluation of bleeding symptoms and assessed within a single protocol.

## 5 Conclusion

In conclusion, similarly to MBD, HMB is highly prevalent among women with BDUC and substantially impairs HMB-related QoL. Women with BDUC and MBD experience more severe menstrual bleeding than those with NBD, and the severity of menstrual bleeding is correlated with the degree of QoL impairment. These findings underscore the need for increased clinical awareness of the impact of HMB in this population, as well as the need for targeted clinical guidelines and optimized management strategies. The use of TXA during menstruation represents an important therapeutic option that could be more consistently incorporated into the management of HMB among women with BDUC.
