# Supplementary material for: Heavy menstrual bleeding in women with a bleeding disorder of unknown cause: a common symptom impacting daily life functioning
Source: Res Pract Thromb Haemost. 2026 Sep 8;10(6):106950. doi: 10.1016/j.rpth.2026.106950 (PMC13676089; doi:10.1016/j.rpth.2026.106950)
Supplement: Supplementary file 1 [file mmc1.docx]

**Supplementary Table 1. Diagnostic criteria for mild bleeding disorders**

| Disease | Laboratory test | Normal values | Diagnostic criteria |
| --- | --- | --- | --- |
| VWD type 1 | VWF act/ant, ratio | 50-150%, > 0.7 | VWF act and/or ant < 50%, ratio > 0.7 |
| VWD type 2 | VWF act/ant, ratio | 50-150%, > 0.7 | VWF act and/or ant < 50%, ratio < 0.7 |
| VWD type 3 | VWF act/ant | 50-150% | VWF act and/or ant < 5% |
| Platelet function disorder | Light transmission aggregometry | Maximal aggregation > 60%, normal shape of curve | ≥ 2 abnormal aggregations, or abnormal aggregation with ristocetin or collagen 4 µg/ml |
|  | ATP release | COL 1.20-2.79 nmol  EPI 0.86-2.47 nmol  U46619 0.39-1.87 nmol | ≥ 1 clear abnormalities |
| Macrothrombocytopenia | Smear | Normal thrombocytes | Macrothrombocytes |
|  | Thrombocyte count | 150-350 x 10^9/L | Thrombocyte count < 150 x 10^9/L |
| Factor deficiency | Factor II | 60-140% | < 60% |
|  | Factor V | 60-140% | < 60% |
|  | Factor VII | 60-140% | < 60% |
|  | Factor VIII | 50-200% | < 50% |
|  | Factor IX | 60-140% | < 60% |
|  | Factor X | 60-140% | < 60% |
|  | Factor XI | 60-140% | < 60% |
|  | Factor XIII | 70-140% | < 70% |
| Afibrinogenemia | Clottable/immunoreactive fibrinogen, ratio | 1.6-4.2 g/L, 1 | Clottable/immunoreactive fibrinogen undetectable |
| Hypofibrinogenemia | Clottable/immunoreactive fibrinogen, ratio | 1.6-4.2 g/L, 1 | Clottable/immunoreactive fibrinogen < 1 g/L, ratio around 1 |
| Dysfibrinogenemia | Clottable/immunoreactive fibrinogen, ratio | 1.6-4.2 g/L, 1 | Discripancy between clottable and immunoreactive fibrinogen, ratio < 1 |
| Fibrinolysis disorder | Euglobin clotlysis time * | 90-240 minutes | < 60 minutes  < 80 minutes with PAI act < 1.0 |
|  | tPA-ROTEM | Lysis onset time (LOT) > 1560s  Lysis time (LT) > 2100s | LOT < 1560s (in duplo)  LT < 2100s (in duplo) |
|  | PAI-1 activity | < 10.4 ng/mL | - |
|  | PAI-1 antigen | Until 2025 3.4-39 ng/mL  From 2025: 7.1-60 ng/mL | < 3.4 ng/mL with PAI act < 1.0  < 7.1 ng/mL with PAI act < 1.0 |
|  | Α2-antiplasmin activity | 80-130% | < 80% |

******* *Only performed until 2019*

***Abbreviations****: act: activity; ant: antigen; ATP: adenosine triphosphate; COL: collagen; EPI: epinephrine; VWD: von Willebrand disease; VWF: von Willebrand factor.*

*Updated from Heubel-Moenen et al 2022*

**Supplementary Table 2. Domains and statements of the MMAS**

| Domain and statement | Score |
| --- | --- |
| 1. Practical difficulties  1. I have no practical difficulties, bleed no more than I expect and take no extra precautions.  2. I have to carry extra sanitary protection with me but take no other precautions.  3. I have to carry extra sanitary protection and clothes because of the risk of flooding.  4. I have severe problems with flooding, soil the bedding and need to be close to a toilet. | 14.0  9.4  3.1  0 |
| 2. Social life  1. My social life is unaffected during my cycle. I can enjoy life as much as usual.  2. My social life is slightly affected during my cycle. I may enjoy life as much as usual.  3. My social life is limited during my cycle. I rarely make any plans.  4. My social life is devastated during my cycle. I am unable to make any plans. | 10.0  6.7  2.7  0 |
| 3. Psychological health and wellbeing  1. During my cycle I have no worries I can cope normally.  2. During my cycle I experience some anxiety and worry.  3. During my cycle I often feel down and worry about how I’ll cope.  4. During my cycle I feel depressed and cannot cope. | 14.0  8.4  2.9  0 |
| 4. Physical health and wellbeing  1. During my cycle I feel well and relaxed. I am not concerned about my health.  2. During my cycle I feel well most of the time. I am a little concerned about my health.  3. During my cycle I often feel tired and do not feel especially well. I am concerned about my health.  4. During my cycle I feel very tired and do not feel well at all. I am seriously concerned about my health. | 21.0  14.9  4.2  0 |
| 5. Work/daily routine  1. There are no interruptions to my work/daily routine during my cycle.  2. There are occasional disruptions to my work/daily routine during my cycle.  3. There are frequent disruptions to my work/daily routine during my cycle.  4. There are severe disruptions to my work/daily routine during my cycle. | 18.0  11.3  4.1  0 |
| 6. Family life/relationships  1. My family life/relationships are unaffected during my cycle.  2. My family life/relationships suffer some strain during my cycle.  3. My family life/relationships suffer quite a lot during my cycle.  4. My family life/relationships are severely disrupted as a result of my cycle. | 23.0  14.0  5.3  0 |

**Supplementary Table 3. MMAS sensitivity analysis**

|  | BDUC n = 72 | MBD n = 50 | NBD n = 22 | *P* |
| --- | --- | --- | --- | --- |
| MMAS total score | 7.5 [3.0-11.0] | 7.0 [4.0-10.0] | 4.5 [0.0-9.0] | p = 0.220 |
| MMAS subdomain scores  Q1: practical difficulties  Q2: social life  Q3: psychological wellbeing  Q4: physical health  Q5: work/daily routine  Q6: family life | 2.0 [1.0-3.0]  1.0 [0.0-2.0]  1.0 [0.0-2.0]  2.0 [1.0-2.0]  1.0 [0.0-2.0]  1.0 [0.0-1.0] | 1.5 [1.0-2.0]  1.0 [0.0-2.0]  1.0 [0.0-2.0]  2.0 [1.0-2.0]  1.0 [0.0-2.0]   1.0 [0.0-1.0] | 1.0 [0.0-2.0]  1.0 [0.0-1.0]  0.5 [0.0-2.0]  1.0 [0.0-2.0]  0.0 [0.0-1.0]  0.0 [0.0-2.0] | p = 0.029  p = 0.319  p = 0.820  p = 0.770  p = 0.097  p = 0.763 |

*Data are presented as median [IQR]*

*For this analysis, the weighted scores from the original MMAS validation study were replaced with proportional scores. The statements within each subdomain were assigned an equal score ranging from 0 (statement 1) to 3 (statement 4), resulting in a maximum total score of 18, where higher scores indicate poorer HMB related QoL. This differs from the original MMAS score system, in which higher scores indicate better quality of life.* ***Abbreviations****: MMAS: Menorrhagia Multi-Attribute Scale; BDUC: Bleeding Disorder of Unknown Cause; MBD: Mild Bleeding Disorder; NBD: No Bleeding Disord**er*

**Supplementary Table 4. MMAS score in women with and without HMB with HMB, using an ISTH-BAT menorrhagia subdomain score of ≥2 as HMB threshold**

|  | | Number | MMAS score | *P* |
| --- | --- | --- | --- | --- |
| BDUC | **HMB** | 49 | 42.2 [31.0 – 69.2] | p = 0.002 |
|  | **No HMB** | 23 | 70.0 [54.3 – 95.4] |  |
| MBD | **HMB** | 33 | 45.9 [45.9 – 59.2] | p = 0.005 |
|  | **No HMB** | 17 | 74.7 [63.0 – 95.4] |  |
| NBD | **HMB** | 9 | 46.1 [5.8 – 71.9] | p = 0.014 |
|  | **No HMB** | 13 | 86.5 [69.2 – 100.0] |  |

*Data are presented as median [IQR]*

***Abbreviations****: MMAS: Menorrhagia Multi-Attribute Scale; BDUC: Bleeding Disorder of Unknown Cause; MBD: Mild Bleeding Disorder; NBD: No Bleeding Disorder; HMB: Heavy Menstrual Bleeding.*

**Supplementary Table 5. Effect HMB and disease group on total MMAS scores**

| Effect | df | F | *p* |
| --- | --- | --- | --- |
| HMB | 1, 138 | 36.74 | p = < 0.001 |
| Disease group | 2, 138 | 0.12 | p = 0.884 |
| HMB x disease group | 2, 138 | 0.56 | p = 0.574 |

*Analysis performed with two-way ANOVA.* ***Abbreviations:*** *HMB: Heavy Menstrual Bleeding*
